# 

**Published:** 1883-03

**Authors:** 


### CONTENTS.

ORIGINAL:

Some Facts About Feeble-Minded Children, and their Care and Training in State Institutions. By Isaac N. Kerlin, M.D. .... 321

A Case of Cerebro-Spinal Meningitis, or Cerebro-Spinal Fever—Its Diagnosis, Circumstances of its Origin, and Treatment. By John H. Logan, M.D. .... 329

An Unusual Case of Hæmaturia. By P. R. Cortelyou, M.D. .... 332

SOCIETIES:

The Atlanta Medical Society—Simulated Stricture of the Œsophagus—Bright's Disease—Typhoid Fever with Intestinal Hemorrhage and Relapse—Septicæmia. .... 334-338

EXTRACTS:

Infantile Paralysis—Best Methods of Treating Operative Wounds—Styptics in General Surgery—Suppurating Synovitis of the Knee—Treatment of Partial Trichiasis by Electrolysis—Urticaria—Treatment of Icterus Neonatorum—Quackery in America—Removal of a Vesical Calculus Weighing Three Thousand Five Hundred and Forty-one Grains from a Boy Sixteen Years of Age—Tonsilotomy—Pop-Corn in the Vomiting of Pregnancy—Syphilis of the Spinal Cord—The Dangers of Amateur Doctoring—Artificial Illustrations of Percussion Sounds—Spray in Abdominal Surgery—Chloroform Water—Mistake by a Druggist; Damages—The Quintescence of Absurdity—Darwin's Charity..338-368

EDITORIAL:

The State Board of Health of Illinois—An Example Worthy of Imitation .... 369

The Critics of the Committee on Revision of the New Pharmacopœia .... 371

Our Annual Volume of Transactions .... 372

The State Medical Society of New York and the New Code. .... 373

Correspondance .... 374

BOOKS. .... 379

PAMPHLETS .... 383

ITEMS .... 383

